# Molecular typing of multi-drug resistant *Acinetobacter baumannii* isolates from clinical and environmental specimens in three Iranian hospitals by pulsed field gel electrophoresis

**DOI:** 10.1186/s12866-020-01792-w

**Published:** 2020-04-25

**Authors:** Ali Mohammadi Bardbari, Parviz Mohajeri, Mohammad Reza Arabestani, Manoochehr Karami, Fariba Keramat, Saba Asadollahi, Amir Khodavirdipour, Mohammad Yousef Alikhani

**Affiliations:** 1 Department of Microbiology, Faculty of Medicine, Hamadan University of Medical Sciences, Hamadan, Iran; 2 Department of Microbiology, Faculty of Medicine, Kermanshah University of Medical Sciences, kermanshah, Iran; 3 Department of Epidemiology, School of Public Health, Hamadan University of Medical Sciences, Hamadan, Iran; 4 Department of Infectious Diseases, Faculty of Medicine, Hamadan University of Medical Sciences, Hamadan, Iran; 5 Brucellosis Research Center, Hamadan University of Medical Sciences, Hamadan, Iran; 6 Division of Human Genetics, Department of Anatomy, St. John’s Hospital, Bangalore, India

**Keywords:** *A.baumannii*, MDR, Biofilm formation, Antibiotic resistance, PFGE

## Abstract

**Background:**

Multi-drug resistant (MDR) *Acinetobacter baumannii* is one of the most important causes of nosocomial infections. The purpose of this study was to identify antibiotic resistance patterns, biofilm formation and the clonal relationship of clinical and environmental isolates of *A. baumannii* by Pulsed Field Gel Electrophoresis method. Forty-three clinical and 26 environmental isolates of the MDR *A. baumannii* were collected and recognized via API 20NE. Antibiotic resistance of the isolates was assessed by the disk diffusion method, and the biofilm formation test was done by the microtiter plate method. Pulsed Field Gel Electrophoresis (PFGE) was used to assess the genomic features of the bacterial isolates.

**Results:**

The resistance rate of clinical and environmental isolates against antibiotics were from 95 to 100%. The difference in antibiotic resistance rates between clinical and environmental isolates was not statistically significant (*p* > 0.05). Biofilm production capabilities revealed that 31 (44.9%), and 30 (43.5%) isolates had strong and moderate biofilm producer activity, respectively. PFGE typing exhibited eight different clusters (A, B, C, D, E, F, G, and H) with two significant clusters included A and G with 21 (30.4%) and 16 (23.2%) members respectively, which comprises up to 53.6% of all isolates. There was no relationship between biofilm formation and antibiotic resistance patterns with PFGE pulsotypes.

**Conclusions:**

The results show that there is a close relationship between environmental and clinical isolates of *A. baumannii*. Cross-contamination is also very important that occurs through daily clinical activities between environmental and clinical isolates. Therefore, in order to reduce the clonal contamination of MDR *A. baumannii* environmental and clinical isolates, it is necessary to use strict infection control strategies.

## Background

*Acinetobacter baumannii* is a gram-negative bacterium and one of the important pathogens of nosocomial infections, including pneumonia, meningitis, bacteremia, urinary tract infections, surgical wounds and soft tissue infections [1]. It has a key role in worldwide nosocomial infections, especially in the adult intensive care units (ICUs) [2, 3]. Due to numerous factors, including prolonged hospital admission, loss of the skin barrier, and complex treatment protocols, patients admitted to ICU wards are significantly susceptible to nosocomial infections [4].

Recently, due to the use of broad-spectrum antibiotics, antimicrobial resistance between *A. baumannii* isolates has increased significantly. Therefore, the emergence of multi-drug resistant (MDR) and extensively drug-resistant (XDR) *A. baumannii* isolates as an important cause of nosocomial infections is one of the major health problems in different countries of the world [2, 5, 6]. The impervious outer membrane and environmental exposure to a large pool of resistance genes are considered as selective pressures that cause XDR isolates in these bacteria [7]. This pathogen possesses a remarkable ability to survive and widely spreading in hospital environments and mucosal surfaces [8]. Long-term survival is likely to be a major cause of hospital transmission of this organism, especially in ICU wards and through healthcare staff [1]. For this reason, particular attention has been paid to the capability of *A. baumannii* to cause outbreaks of nosocomial infections and to obtain resistance to antibiotics [4]. The ability of *A. baumannii* to form biofilms on living and non-living surfaces is an important factor in the persistence of bacteria because it protects them against environmental stress conditions, such as desiccation and exposure to antibiotics and disinfectants, which makes biofilm infections persistent and challenging to treat [9]. For epidemiological studies, several typing methods have been used to investigate outbreaks caused by *A. baumannii*. The usually applied methods focus on differences in the phenotypic properties that have insufficient reproducibility and discriminatory power. Molecular approaches such as PFGE that compare the DNA differences of bacteria have been accepted because of establishing the clonal association in many bacteria including *A. baumannii* isolates [10]. So far, few investigations have been done on the relationship between environmental and clinical isolates of *A. baumannii* in patients admitted to intensive care units. Concurrent typing of clinical and environmental isolates of *A. baumannii* is an important tool for finding sources and ways of transmission of such epidemic isolates. This research aimed to identify antibiotic resistance patterns, biofilm formation and clonal association of clinical and environmental isolates of *A. baumannii* by PFGE technique.

## Results

### Susceptibility to antibiotics

**Fig. 1:**
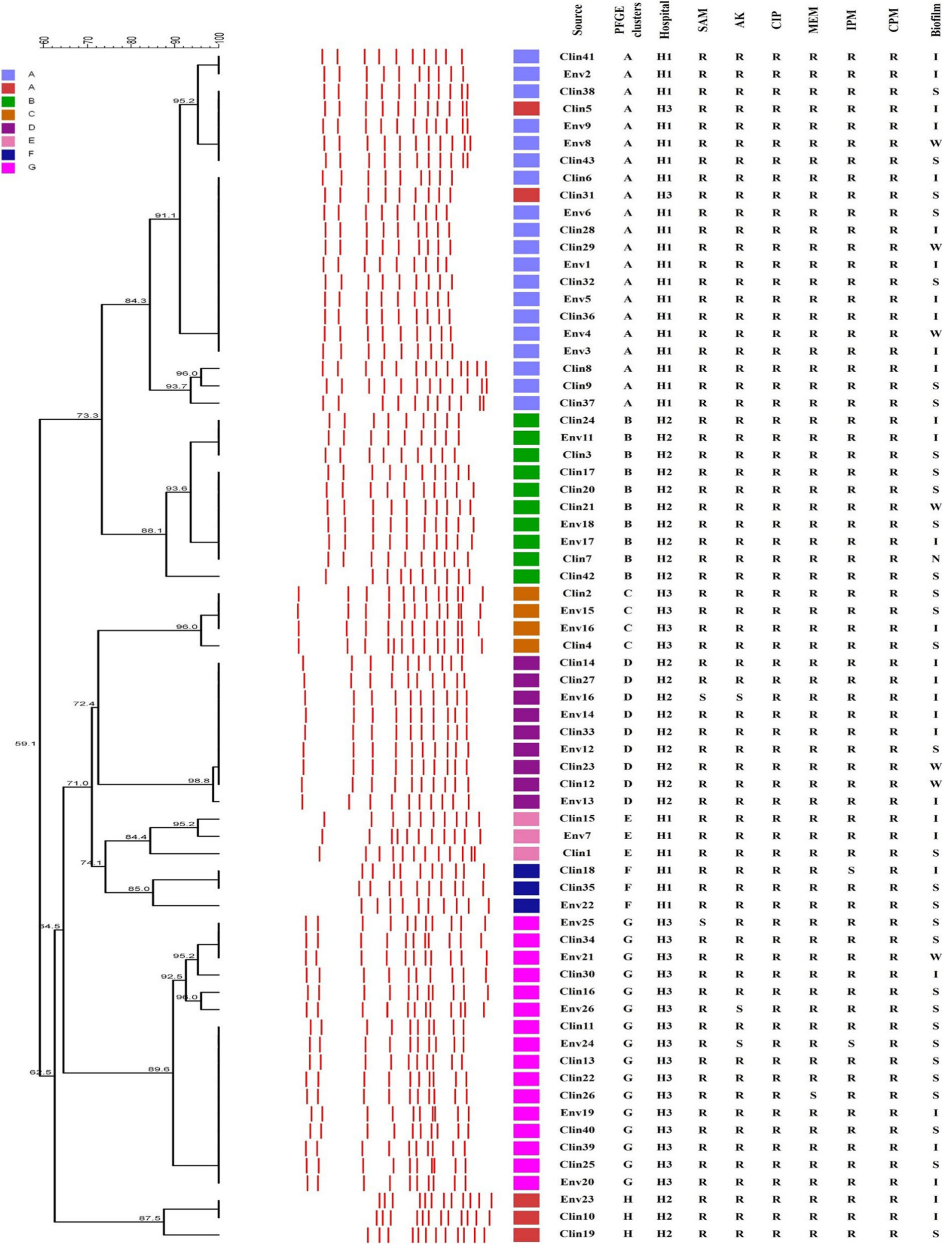
Dendrogram cluster analysis of PFGE data for clinical and environmental *A.baumannii* isolates. Clin; clinical, Env; environmental, H1; Besat hospital, H2; Behashti hospital, H3; Sina hospital, R; resistance, SAM; ampicillin-sulbactam, AK; amikacin, CIP; ciprofloxacin, MEM; meropenem, IPM; imipenem, CPM; cefepime, N; non-biofilm producer, W; weak, I; intermediate, and S; strong biofilm producer

The results of the antimicrobial susceptibility test shown in Fig. 1. All clinical and environmental isolates of *A. baumannii* (100%) were susceptible to colistin and tigecycline and all isolates (100%) were resistant to ciprofloxacin and cefepime. The resistance rate against ampicillin-sulbactam, meropenem, imipenem, and amikacin in the clinical isolates were 43(100%), 42(97.7%), 42(97.7%), 43(100%) and in environmental isolates were 24(92.3%), 26(100%), 25(96.2%), and 23(88.5%), respectively. Most clinical (95.3%) and environmental (84.6%) isolates of *A. baumannii* were resistant to all tested antibiotics and designated as extensively drug-resistance (XDR). The difference in antibiotic resistance rates between clinical and environmental isolates was not statistically significant (*p* > 0.05).

### Biofilm formation

One of the major virulence-related features of *A. baumannii* is the ability of biofilm formation. Therefore, we decided to measure potential biofilm formation in XDR of clinical and environmental isolates. In our study, we found that 68 (98.6%) of the isolates were capable of forming biofilm. The mean OD 595 values for clinical and environmental isolates were 0.680 ± 0.289 and 0.540 ± 0.265, respectively. Biofilm production capabilities revealed that 31 (44.9%), 30 (43.5%), 7 (10.2%), and 1 (1.4%) isolates had strong, moderate, weak, and no biofilm producer activity in the microplate assay, respectively. No statistically significant difference in biofilm formation was seen among the clinical and the environmental isolates (*p* > 0.05).

### PFGE results

The PFGE method by *ApaI* enzyme was used for access typing and genetic relationship between the *A.baumannii* isolated from clinical and environmental samples. From 26 environmental and 43 clinical isolates, 8 common PFGE clusters (A, B, C, D, E, F, G, and H were obtained (Fig. 1). The similar strains in two hospital were seen. PFGE clusters A, E, and F were seen in the clinical and environmental isolates that recovered from Besat hospital. PFGE cluster A was the predominant clones with 21 members, which of them, 13 members isolated from patients and 8 from environmental surfaces (Table 1).

**Table 1:** The frequency distribution of *A. baumannii* in clinical and environmental isolates based on the location of specimen collection and PFGE type

Pattern	Clinical isolates No (%)	Environmental isolates No (%)	Total No (%)	Status of pulsotypes
A	13 (30.2)	8 (30.8)	21(30.4)	Major pulsotype
B	7 (16.3)	3 (11.5)	10 (14.5)	Intermediate pulsotype
C	2 (4.6)	2 (7.7)	4 (5.8)	Minor pulsotype
D	5 (11.6)	4 (15.4)	9 (13.0)	Intermediate pulsotype
E	2 (4.6)	1 (3.8)	3 (4.3)	Minor pulsotype
F	2 (4.6)	1 (3.8)	3 (4.3)	Minor pulsotype
G	10 (23.3)	6 (23.1)	16 (23.2)	Major pulsotype
H	2 (4.6)	1 (3.8)	3 (4.3)	Minor pulsotype
Total	43(100)	26(100)	69(100)	–

## Discussion

*Acinetobacter baumannii* is becoming an increasingly well-known pathogen because of the increase in the number of infections caused by this organism and the development of MDR and XDR strains [11]. The potential of *A. baumannii* to persist in either moist or dry conditions in the hospital environment is a consequence of the presence of multiple antibacterial resistance genes and biofilm formation makes this bacterium a successful pathogen among nosocomial bacteria [12]. The unique ability of this bacterium to survive in the environment for a long time demonstrates its role in the outbreaks of nosocomial infections [13]. Contaminated environmental surfaces can contribute directly to the transmission of pathogens to patients or from the hands of health care workers to patients [14].

The results of this study show high environmental pollution in the three intensive care units in our area. The incidence of XDR *A. baumannii* isolated from environmental surfaces 22(84.6%) which, were resistant to all tested antibiotics was greater than that detected in previous studies in Germany (7.3%), United States (9.8%), and 13.1% in China [1, 13, 15]. These results can probably be attributed to inappropriate strategies of disinfection and hand washing by health workers in hospitals. Furthermore, 41(95.3%) clinical isolates were resistant to all tested antibiotics and were XDR, which agrees with other investigations conducted in Iran [16, 17]. Of the 8 antimicrobials tested, the most potent ones were colistin and tigecycline (100%) for all clinical and environmental isolates. In agreement with previously research who requested, the most effective drug in controlling *A. baumannii* is polymyxin B [18].

Of the 41 XDR *A. baumannii* strains isolated from patients’ respiratory tracts, the bacteria isolated from tracheal aspirate specimens were the most common respiratory isolates, which is consistent with previous studies [19, 20]. Consistent with the earlier study, the resistance rate of clinical and environmental isolates of *A. baumannii* to antibiotics was 95–100% and there was no significant difference between antibiotic resistance in clinical and environmental isolates (*p* > 0.05) [21]. One of the important features related to the virulence of *A. baumannii* is its ability to form biofilms. In our study, we determined that 68 (98.6%) isolates of XDR *A. baumannii* formed biofilm, which is in agreement with previous studies [2, 17, 22]. According to our results, 44.9% of isolates showed strong ability to biofilm formation. Our results are consistent with previous reports which showed that more than 75% of *A. baumannii* isolates form biofilms [23, 24]. Previous studies have reported a positive relationship between biofilm formation and antibiotic resistance in *A. baumannii* isolates [17, 25, 26]. In our study, all strong biofilm forming *A. baumannii* isolates were XDR.

To track and evaluate the outbreaks, the genetic association of the isolates, and to attribute one strain to the relevant clonal lineage, several molecular typing techniques have been developed [27, 28]. Among these methods, PFGE is considered the gold standard due to its discriminatory power, reproducibility, and sensitivity, and to determine the single-colonal pattern of hospital outbreaks, the prevalence of pathogens within and between hospitals and their stability in the environment are used [28].

In the current study typing of XDR *A. baumannii* isolates was done for tracks of outbreak and analyses of a population survey of bacteria based on their genotypes, predominant genotypes, distribution and probability transmission of isolates between patient and environmental surfaces. By the PFGE technique, 43 clinical and 26 environmental *A. baumannii* isolates were typed. PFGE typing showed 8 different PFGE cluster (A, B, C, D, E, F, G, and H) with two major cluster A and G with 21 (30.4%) and 6 (23.2%) members, respectively, which contains up to 53.6% of all isolates. In our study, a close genetic relationship between clinical and environmental isolates of *A. baumannii* was observed that is consistent with other studies [4, 21]. These results indicate that the hospital environment is frequently colonized by different *A. baumannii* clones, which may be responsible for the transmission of *A. baumannii* isolates between patients and their surroundings.

In our study, two clinical isolates (No 5 and 31) which were found in two distinct hospitals were clustered into pulsotype A. This issue may indicate the possible transfer of related isolates from one ICU to another in the same hospital or different hospitals from patients admitted to the ICUs or the hospital health team in the same city. This type of transmission has been reported in several countries [2, 29]. Comparing the frequency of biofilm formation ability in clinical and environmental isolates with pulsotypes, no significant correlation was found, which is consistent with the study of Wroblewska et al. [30].

In our study, the correlation analysis of PFGE typing and antibiotic resistance profiles showed that most isolates were XDR and no difference in antibiotic resistance was found in the PFGE clusters. Therefore, there is no significant relationship between different PFGE clusters and antimicrobial resistance patterns. This indicates that antimicrobial resistance patterns have low discriminatory power for bacterial typing and highlights the necessity of genotyping techniques such as PFGE to categorize isolates with similar phenotypes and distinct genetic relatedness during the evaluation of outbreak episodes or horizontal transmission of isolates in the hospital environments [31].

## Conclusions

Our investigation shown the high frequency of biofilm forming XDR *A. baumannii* with a high prevalence of biofilm formation. Tracing the sources of environmental isolates indicates that there is a close genetic link between environmental and clinical isolates of *A. baumannii*. Besides, it suggests that the occurrence of cross-contamination events is likely to occur between environmental and clinical isolates during routine clinical activities. Therefore, the use of strict infection control strategies to reduce cross-contamination of endemic clones of *A. baumannii* isolates is essential.

## Methods

### Bacterial isolates

In this cross-sectional study, 43 MDR *A. baumannii* were collected from respiratory tracts of patients admitted to ICU wards of Besat, Sina, and Beheshti educational hospitals of Hamadan University of Medical Sciences in Hamadan, west of Iran, during a period between November 2015 and August 2016.

The Besat hospital is a major tertiary referral hospital where patients are referred from neighboring provinces and Sina and Beheshti hospitals have infectious and internal medicine departments respectively, which accept patients in Hamadan province. Simultaneously, 26 MDR *A. baumannii* strains isolated from different environments and equipment surfaces such as ventilators, sink, and ground, hands of Staff, trolleys, bedside table, pillow and linens. For sampling from the environment and equipment of ICU wards, an area of about 10 cm2 was selected and sampled using a sterile humidified swab with physiological serum.

### Culture and identification

After taking the samples, the swabs were inoculated in Brian heart infusion broth (BHI) media and incubated overnight at 35 °C and further subcultured on MacConkey’s agar plates at 37 °C for 24 h. The *Acinetobacter* spp. were identified by colony morphology, growth at 44 °C, oxidase, OF (Oxidation and fermentation), Simon citrate, and API 20NE system (BioMérieux Co, France). The *A. baumannii* isolates identification was confirmed by PCR of the blaOXA-51 gene. *A. baumannii* ATTC 19606 was used as a reference strain [32].

### Antibacterial susceptibility test

Antimicrobial susceptibility test was accomplished by the Kirby-Bauer disk diffusion method using the ampicillin/sulbactam (10 μg /10 μg), imipenem (10 μg), meropenem (10 μg), amikacin (30 μg), cefepime (30 μg), colistin (10 μg), tigecycline (15 μg), and ciprofloxacin (5 μg), antibiotic disks (Mast Group Co, UK). The results interpreted according to Clinical and Laboratory Standard Institute guidelines (CLSI) [33]. *Pseudomonas aeruginosa* ATCC 27853 was used as a control strain. MDR *A. baumannii* isolates were defined as resistant to three or more classes of antibiotics as previously described [34].

### Biofilm assay

The ability of *A. baumannii* isolates to produce biofilm was assessed by the microtiter plate method as previously described [35]. Briefly, biofilm formation was performed in triplicate from overnight cultures diluted in Tryptic soy broth (TSB) medium supplied with 1% glucose to an optical density (OD) of 0.01 at 600 nm and deposited in 96-well plates. TSB medium without inoculum was used as a negative control. The plate was incubated at 37 °C for 24 h with gentle shaking. The wells were washed three times with Phosphate Buffer Saline (PBS) solution. Absolute methanol was added per well to biofilm fixation. Biofilm was stained with 1% crystal violet (w/v) and quantified at 595 nm after solubilization with absolute ethanol for 15 min at room temperature. Biofilm production was interpreted according to the criteria of Stepannovic et al. [36]. The optical density cut-off value (ODc) was established as three standard deviations (SD) above the mean of the optical density (OD) of the negative control as showed in the following formula: ODc = average OD of negative control+(3 SD of negative control).

The results were divided into the four following categories according to their optical densities as strong biofilm producer (4 ODc < OD); medium biofilm producer (2 ODc < OD ≤ 4 ODc); weak biofilm producer (ODc < OD ≤ 2 ODc); and non-biofilm (OD ≤ ODc) [36].

### Pulsed-field gel electrophoresis typing

Genetic similarities among clinical and environmental isolates of *A. baumannii* were investigated by PFGE as previously described [37]. Briefly, an overnight culture of bacteria was suspended in 100 μl of cell suspension buffer and was mixed with an identical volume of 2% low melting agarose and distributed in a plug mold. Genomic DNA in agarose plugs was lysed in the cell lysis solutions I and II, washed and digested with ApaI restriction enzyme (Thermo Scientific, USA). The Lambda PFG Ladder (New England, Biolabs) was used as a DNA size marker. Electrophoresis of digested DNA was performed in a pulsed-field electrophoresis system (Chef Mapper; Bio-Rad Laboratories, USA) by programming two states with the following conditions: temperature 14 °C; voltage 6 V/cm; switch angle, 120°; switch ramp 2.2–35 s for 19 h.

### Cluster analysis

Gel images were studied by BioNumerics software version 7.5 (Applied Maths, StMartens-.

Latem, Belgium). Dendrograms were obtained for all of the isolates. A comparison of the banding patterns was done by the unweighted pair group method with mathematical averaging (UPGMA), and DNA similarity was considered by using the band-based Dice coefficient with a tolerance setting of 1.5% band tolerance and 1.5% optimization setting were applied during comparison of the DNA patterns. The PFGE results were compared according to the criteria by Tenover et al.; a PFGE cluster was based on a similarity cutoff of 80% [38].

### Statistical analysis

Statistical analysis was performed using SPSS 23.0 (SPSS, Chicago, IL, USA). The frequency of susceptibility and biofilm formation category were determined in clinical and environmental isolates. The relationship among biofilm formation and the antibiotic resistance with PFGE type were made using chi-square tests. A *P*-value of less than 0.05 was considered as statistically significant.

## Data Availability

The datasets used and/or analyzed during the current study available from the corresponding author on reasonable request.

## Abbreviations

MDR: Multidrug-resistant
PFGE: Pulsed Field Gel Electrophoresis
ICUs: Intensive care units
XDR: Extensively drug resistant
CLSI: Clinical and Laboratory Standard Institute guidelines
TSB: Tryptic soy broth
OD: Optical density
PBS: Phosphate Buffer Saline
